# FLIM-Phasor Analysis (FLIM-ϕ) of Aβ-Induced Membrane Order Alterations: Towards a Cell-Based Biosensor for Early Alzheimer’s Disease Diagnosis

**DOI:** 10.3390/mi16020234

**Published:** 2025-02-19

**Authors:** Antonella Battisti, Maria Grazia Ortore, Silvia Vilasi, Antonella Sgarbossa

**Affiliations:** 1 NEST, Nanoscience Institute-CNR and Scuola Normale Superiore, p.zza San Silvestro 12, I-56127 Pisa, Italy; antonella.sgarbossa@nano.cnr.it; 2 Biophysics Institute-CNR, Via Ugo La Malfa 153, I-90146 Palermo, Italy; silvia.vilasi@cnr.it; 3 Dipartimento di Scienze Della Vita e dell’Ambiente, Università Politecnica delle Marche, Via Brecce Bianche, I-60131 Ancona, Italy; m.g.ortore@univpm.it

**Keywords:** Alzheimer’s disease, cell-based biosensor, amyloid peptide biosensor, FLIM-phasor analysis, Aβ detection

## Abstract

Alzheimer’s disease (AD) is a progressive neurodegenerative disorder, and its early detection can be critical for a prompt intervention that can potentially slow down the disease progression and improve the patient’s quality of life. However, a diagnosis based solely on clinical symptoms can be challenging, especially in the early stages, while the detection of specific biomarkers such as amyloid-β peptide (Aβ) and tau proteins can provide objective evidence for diagnosis. In this work, we explored the effects of Aβ peptide on cell membrane properties thanks to fluorescence lifetime imaging (FLIM) combined with the phasor analysis (FLIM-ϕ). The results showed that the membrane viscosity is altered by the presence of Aβ peptide and that cells experience this effect even at nanomolar concentrations of peptide. This considerable sensitivity opens up the possibility of envisioning a cell-based biosensor able to detect very low concentrations of Aβ in a biological fluid, thus enabling timely diagnosis and intervention.

## 1. Introduction

Alzheimer’s disease (AD) is a degenerative disorder of the central nervous system that manifests with memory disturbances, deficits in abstract thinking, personality and behavioral changes, and progressive cognitive decline, leading to an inability to perform normal daily activities. It is currently considered the most common cause of senile dementia, and it can affect individuals regardless of their nationality, race, ethnic group, or social status, although the incidence may vary across different categories [1]. Current research focuses on understanding the possible causes that may trigger the onset of the disease, such as accelerated aging, genetic predisposition, and both environmental and individual toxic factors.

One of the pathological markers of AD is the amyloid-β peptide (Aβ), a 40–42 amino acid peptide that accumulates in insoluble and organized plaques in the AD-affected brain. Historically, the method of choice for assessing Aβ levels has been cerebrospinal fluid (CSF) analysis. Specifically, the ratio of Aβ42 to Aβ40 in CSF is considered a robust biomarker, since decreased Aβ42 levels and a consequently altered Aβ42/Aβ40 ratio reflect amyloid plaque deposition within the brain parenchyma [2,3]. This method, although efficient, is highly invasive, thus motivating the development of less invasive assays, such as blood-based assays. These detection methods, while promising and more acceptable, face significant challenges, including the considerably lower concentrations of Aβ in blood than in CSF, as well as the potential influence of peripheral Aβ production [4]. Moreover, such methods must be standardized and correlated with both CSF Aβ levels and positron emission tomography (PET) imaging, the current gold standard for in vivo amyloid imaging, that recently has also been coupled to deep learning algorithms for the evaluation of minimally processed brain PET scans [5]. And critically, Aβ is only seldom employed as a unique indicator for the diagnosis of AD, but rather integrated with other established markers, such as tau protein (another key pathological marker), neuroimaging findings (e.g., hippocampal atrophy), and, crucially, a thorough clinical assessment encompassing cognitive testing and neuropsychological evaluation, whose alterations usually have a late onset [6,7]. The refinement of the available Aβ detection systems (relying, e.g., on single-molecule detection systems [8], liquid crystals [9,10], electrochemistry [11,12], mass spectrometry [13], fluorescence [14], etc.), along with multiplex detection of Aβ and other circulating biomarkers, could lead to a more accurate and accessible diagnostic paradigm for AD [15]. This implies expanding the arsenal of Aβ revelation methods focusing on improving their performance and exploring Aβ biological activity in order to find new ways to detect its presence.

Aβ derives from a specific proteolytic pathway of a ubiquitously expressed transmembrane glycoprotein known as amyloid precursor protein (APP). This protein can undergo sequential cleavage by β- and γ-secretases, releasing the 40- or 42-amino acid peptide. When the production of Aβ peptide increases or its clearance decreases, it triggers what is commonly referred to as the “amyloid cascade”, a series of events that begins with peptide accumulation and leads to progressive neuronal dysfunction culminating in the established pathology [16]. Aβ undergoes a self-aggregation process that involves the formation of various species, ranging from low-nucleation oligomers to large aggregates. These aggregates can sometimes be amorphous, but more often, they exhibit highly organized polymorphic fibrillar structures [17,18,19] both in vitro and when formed inside the human brain. These fibrils are non-branched filaments, several microns long, formed by the winding of thick protofilaments (2–5 nm). They have a high percentage of β structure forming antiparallel β-sheets in a cross-β arrangement characteristic of all amyloid fibers, and they are insoluble and highly resistant to proteases, making them difficult to clear once formed. Additionally, they tend to aggregate into large plaques in the extracellular matrix.

In the literature, the aggregation of amyloid peptides is described according to a nucleation–polymerization and fragmentation kinetic model, where each phase can be characterized by specific structural intermediates with varying sizes, morphologies, and cytotoxic potential. The formation of mature fibers is preceded by peptide assembly into various metastable non-fibrillar species, known as prefibrillar aggregates [20]. However, the cytotoxic potential looks greater for low-nucleation oligomers than for the mature fibrils [21]. Studies on simple cellular systems have highlighted the fundamental role played by soluble oligomeric species of Aβ in cell death, a convergence point for all neurodegeneration mechanisms. These oligomers, whose dimensions do not exceed a few nanometers and whose secondary structure is less ordered than that of fibrils, can interact with cell membranes, disrupting cellular metabolic balance and triggering programmed events that lead to neuronal death. Soluble oligomers derived from other amyloidogenic proteins seem to adopt the same “toxic fold” conformation as the Aβ peptide, exerting a common mechanism of toxicity where the plasma membrane plays a critical role. On the one hand, the cytotoxic effect results from direct Aβ/membrane interaction in target cells, involving changes in calcium homeostasis in membrane channels [22] and the production of reactive oxygen species (ROS) due to membrane oxidation [23]. On the other hand, parameters related to the mechanical state of the membrane, such as permeability and integrity, can be affected and then they can serve as indicators of the cell’s physiological status. Recently, in a mouse model (Tg2576) of Alzheimer’s disease, it was shown that the interaction between Aβ and the membrane depends to some extent on the degree of order in the lipid bilayer. Lipid rafts in the plasma membrane serve as sites where Aβ oligomers accumulate, contributing to severe cytotoxic mechanisms in AD. These rafts, which are highly ordered and rigid membrane zones, appear to act as aggregation centers for Aβ [24].

Confocal fluorescence microscopy has been used to investigate the behavior of cellular membranes in the presence of Aβ in model systems [25]. Thanks to fluorescent probes able to stain the lipid membrane and to discriminate between ordered and disordered phases, significant information about the interaction mechanism between the peptide and the membrane can be collected. In this context, fluorescence lifetime imaging (FLIM) coupled to the phasor analysis (FLIM-ϕ) allows for a fit-free, concentration-independent measurement requiring only a few hundreds of photons per pixel [26].

FLIM-ϕ relies on the luminescence of fluorophores that can be designed in terms of chemical and photophysical properties to stain desired features and to react to different stimuli, with high sensitivity (down to single molecule) and multiplexing ability, as well as specific susceptibilities to environmental changes in the system pH [27,28], viscosity [29,30,31], polarity [32,33], ionic content [34,35], hydration [36,37], solvation [38,39], mechanical stress [40,41], phase behavior [42], signaling [43,44,45], and even combinations of parameters that can be relevant during chemical or biochemical reactions [46,47,48].

In this work, the fluorescent probe Ge1L, which owns a chemical structure inspired by the GFP chromophore, a membrane-targeting lipid moiety and distinct fluorescence lifetimes when distributed within lipid ordered (L_o_) or disordered (L_d_) phases [49,50,51], has been tested under FLIM-ϕ to quantify alterations in cell membrane order upon exposure to moderate concentrations of Aβ.

## 2. Materials and Methods

### 2.1. Aβ Oligomers (Aβ-Olig) and Fibrils (Aβ-Fib) Preparation

The lyophilized synthetic peptide Aβ_1–40_ (Biopeptide Co., Inc., San Diego, CA, USA) was solubilized in NaOH 2 mM, pH 10, sonicated and lyophilized according to a reference protocol [52]. To obtain a solution containing only monomers or small oligomers (Aβ-olig) with 2–5 peptide units, the lyophilized peptide was re-dissolved in phosphate-buffered solution (PBS, pH 7.4) and then filtered through a 0.20 μm filter to eliminate large aggregates, following a well-established protocol developed in our laboratories [53]. The global concentration of Aβ in Aβ-olig was assessed by spectroscopic measurement of tyrosine absorption at 276 nm using an extinction coefficient of 1390 cm^−1^ M^−1^. To obtain amyloid fibrils-containing solutions (Aβ-fib), aggregation was induced by incubation of 75 μM Aβ_1–40_ in 0.1 M PBS (pH 7.4) for 6 h at 45 °C under magnetic stirring in the thermostatic cell holder of a Fluoromax-4 spectrofluorometer (Horiba Jobin Yvon, Longjumeau, France). The aggregation process was monitored by light-scattering measurements at 90° (Supplementary Materials, Figure S1) by setting both the excitation and the emission monochromators at 405 nm [54]. Mature fibrils were stained with 5 μM ThT and the resulting fluorescence was measured with the abovementioned fluorometer (λ_exc_ = 442 nm, Figure S2 in the Supplementary Materials). Stained fibrils were also imaged by confocal fluorescence microscopy using a Leica TCS SP5 inverted laser scanning confocal microscope (Leica Microsystems AG, Wetzlar, Germany) equipped with internal Ar laser for excitation at 458 nm (Supplementary Materials, Figure S3).

### 2.2. Cell Culture

U2OS cells were grown in Dulbecco’s modified Eagle medium: F-12 nutrient mix (DMEM/F-12) purchased from Invitrogen (Carlsbad, CA, USA) supplemented with 10% fetal bovine serum and 100 U/mL penicillin, and 100 mg/mL streptomycin (Invitrogen). Cells were maintained at 37 °C in a humidified 5% CO_2_ atmosphere. For FLIM-ϕ, 120,000 cells were plated onto a 35 mm glass-bottom dish (WillCo-dish GWSt-3522, WillCo Wells B.V., Amsterdam, The Netherlands) and incubated with 0.3 µL of Ge1L (provided courtesy of authors of Ref. [49]) 1 mg/mL in DMEM for 15 min before Aβ administration. Aβ-olig or Aβ-fib solutions were administered to cells at 37 °C under 5% CO_2_ atmosphere and incubated up to 1 h depending on the experiment.

### 2.3. Confocal Microscopy and FLIM-ϕ

Images were obtained using a Leica TCS SP5 inverted laser scanning confocal micro- scope (Leica Microsystems AG, Wetzlar, Germany) equipped with an external pulsed diode laser for excitation at 470 nm, a 63 × 1.4 NA oil immersion objective (Leica Microsystems) and a time-correlated single photon counting acquisition card (PicoHarp 300; PicoQuant, Berlin, Germany) connected to internal spectral detectors. Laser repetition rate was set to 40 Hz and acquisition lasted until about 200 photons per pixel were acquired. The line scanning speed was set to 400 Hz, and the wavelength collection range was between 480 and 580 nm. The phasor analysis was performed thanks to the freely available SimFCS software (version 2, https://www.lfd.uci.edu/globals/, accessed on 14 February 2025). Data were elaborated using the embedded fractional analysis calculator in SimFCS.

## 3. Results

The behavior of Ge1L in lipid ordered and disordered phases has been previously characterized in terms of localization, partitioning, and fluorescence (quantum yield, lifetime, anisotropy) [51]. Its lifetime showed great sensitivity towards the degree of order in the lipid phase, and this property can be exploited to quantify the L_o_/L_d_ ratio in the target membrane using FLIM-ϕ. This technique, originally described by Gratton et al. [55] and outlined in the Supplementary Materials, briefly consists of representing in a polar 2D plot (namely the “phasor plot”) the sine (*s_i,j_*) and cosine (*g_i,j_*) Fourier transforms of the normalized fluorescence emission decay *I_i,j_(t)* collected in each pixel (*i*,*j*) of the FLIM image (Equations (S1) and (S2) in the Supplementary Materials). The resulting vector (*s_i,j_*, *g_i,j_*) points on a semicircle of radius ½ for monoexponential decays, and inside the same circle for multiexponential decays. Since the Fourier transform is a linear operator that preserves the addition operation, linear combinations of phasors produced by distinct photophysical states (e.g., fluorescence emission of Ge1L from a blend of L_o_ and L_d_ phases in a given membrane spot) will fall on the segment connecting the phasors of the two individual components, that in this case could be identified as the pure L_o_ and L_d_ phases. These two reference values can be obtained by calibrating the system in multilamellar vesicles showing homogeneous L_o_ or L_d_ phases as described in ref. [51], while the individual contributions of the different lipid phases in the observed membrane spot can be easily quantified by vector operations.

**Figure 1:**
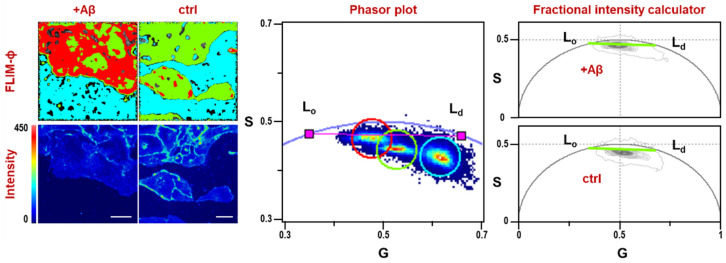
FLIM-ϕ images and phasor plot of Ge1L in U2OS cells. Left: Ge1L in the membrane of U2OS cells with (+Aβ) and without (ctrl) addition of Aβ 100 nM in the medium and intensity images of the selected cells. Scale bar: 20 µm. Center: phasor plot of Ge1L in cells treated with Aβ (red circle), control cells (green circle), reference phasors for Ge1L in L_o_ and L_d_ phases (pink squares) and background emission due to free Ge1L in the medium (cyan circle). Cursor colors in the phasor plot correspond to colors in the FLIM images. Right: fractional intensity calculator showing the calibration (green lines) and phasor clouds given with a minimum threshold set to 20 by the whole set of imaged cells with (top) or without (bottom) Aβ 100 nM.

**Figure 2:**
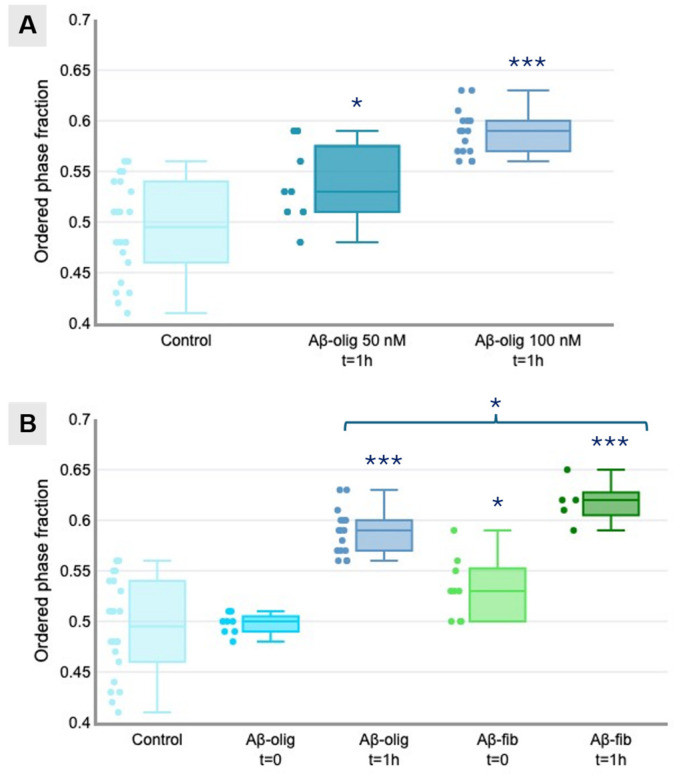
(**A**): L_o_ fraction of control cells and cells treated with 50 nM or 100 nM Aβ-olig for 1 h. (**B**): L_o_ fraction of control cells and cells treated with 100 nM Aβ-olig or Aβ-fib imaged immediately after treatment (t = 0) or after 1 h (t = 1 h).

Images were acquired from untreated cells and from cells treated with solutions of Aβ oligomers (Aβ-olig) 50 or 100 nM and Aβ pre-formed fibrils (Aβ-fib) 100 nM, and the average L_o_ fraction was calculated by vector algebra using the fractional intensity calculator tool in the FLIM-ϕ software. Control cells gave an average L_o_ fraction of 0.50 (Table 1), but deviations from this value have been observed upon treatment with Aβ solutions. To show this effect, the FLIM and intensity images and the phasor plot of two representative samples of treated and untreated cells are reported in the left and center panels of Figure 1. The green circle in the phasor plot encompasses the majority of the pixels relevant to the FLIM image of cells under resting conditions. Upon treatment with Aβ-olig 100 nM, the phasors undergo a shift along the calibration line, toward the L_o_ phase (red circle). Background emission due to free Ge1L in the culture medium has not been filtered out by setting a minimum threshold, because the phasor plot allowed for easy discrimination and isolation of the background pixels (cyan circle); it is worth noting that the pixel clouds deviate from the ideal calibration line due to the contribution of background emission. Conversely, a threshold has been set for fractional intensity calculation for better data visualization in the right panels of Figure 1, where the phasors of the two entire sets of imaged cells (with and without Aβ-olig 100 nM) have been overlapped and plotted together with the calibration line (green lines). The comparison between the two right panels shows how the total pixel cloud given by the whole set of cells treated with Aβ shifts towards an increased ordered fraction. This effect was investigated under different concentration conditions and using different forms of Aβ (oligomers or fibrils). Images belonging to every set of measurements were individually analyzed to calculate the L_o_ fractions, which were finally averaged as reported in Figure 2 and Table 1.

The calculated fractional intensities are reported in Figure 2 as box plots for easy comparison. Figure 2A shows that after one hour of incubation with Aβ-olig 50 nM, a perceptible increase in the degree of membrane order can be observed (*, *p* ≤ 0.05, Student’s *t*-test), and that this degree further increases if incubation is performed with a 100 nM concentration of peptide (***, *p* ≤ 0.001, Student’s *t*-test), reaching an average L_o_ fraction of 0.59.

When the cells are treated with Aβ in the form of fibrils (Figure 2B), a final L_o_ fraction of about 0.62 is reached after 1 h of incubation (***, *p* ≤ 0.001, Student’s *t*-test), which is slightly higher that the final L_o_ fraction obtained with Aβ-olig under the same conditions (*, *p* ≤ 0.05, Student’s *t*-test). However, even immediately after administration of Aβ-fib, a small increment in membrane order is recorded (*, *p* ≤ 0.05, Student’s *t*-test), which is not observed with Aβ-olig. Average calculated L_o_ fractions for cells imaged under different conditions are reported in Table 1.

## 4. Discussion

To enable clinical trials of condition-modifying therapies for AD, which are expected to have the greatest effectiveness at the earliest and mildest stages of the disease, the detection of a preclinical pathological signature is necessary. Aβ_1–40_ aggregates, both oligomer and fibrils, are considered as the major markers for the diagnosis of AD [56,57]. Specifically, plasma amyloid-beta oligomer levels have been proposed as a novel blood-based biomarker for AD prediction [58]. It has been demonstrated that β-sheet-rich Aβ oligomers either permeate the cell membrane or fibrillate on its surface inducing modifications of its mechanical properties [59]. Thus, a cell membrane-based biosensor could be able to detect in body fluids the presence of amyloid structures, indicative of early stages of AD, as recently suggested [9].

The liquid-ordered and liquid-disordered lipid phases are both present and homogeneously distributed in the cellular plasma membrane, contributing to its fluidity and functionality. It has been shown that Aβ peptides tend to localize on the disordered phase [60,61]; however, it is also known that the ordered phase plays a major role in Aβ production, in its assembly into oligomers and in the interaction of oligomers with neuronal receptors [62], thus indicating the existence of a strong link between the peptide behavior and the membrane mechanical properties. This relationship is still being debated since the numerous experiments designed to investigate it are often in disagreement, likely due to the extreme inhomogeneity of experimental conditions in terms of detection technique, peptide preparation and administration, type of membrane or cell, observed time span, etc. It is known that in the long term, the presence of Aβ induces membrane degradation with loss of mechanical rigidity [63]. However, in agreement with several comparable experiments [64,65,66], this work showed that on a timescale of one hour the exposure to nanomolar concentrations of Aβ peptide can increase the membrane stiffness in a concentration- and time-dependent manner. Furthermore, the behavior of Aβ oligomers and fibrils in this context seems to differ to a small extent, with fibrils exerting a slightly more pronounced effect. Likely, Aβ oligomers, being smaller and more soluble, might interact more dynamically with the lipid bilayer, potentially causing transient disruptions, ion channels alterations and localized stiffening. In contrast, Aβ fibrils, which are larger and more structured, could lead to more extensive and stable modifications in membrane order and stiffness. These findings are consistent with previous studies that have highlighted that the peptide assembly state can be correlated to neurotoxicity both in the short- and long-term [67,68], and that soluble and aggregated forms of Aβ interact with different regions of the membrane [63,69]: soluble oligomeric Aβ tends to locate in the hydrophobic core region of the lipid bilayer, whereas aggregated Aβ is more associated with the phospholipid headgroup or hydrophilic area of the membrane. Regardless of the aggregation degree of the peptide, it is clear that the cell membrane is very sensitive to the presence of Aβ that induces a detectable modification on the membrane order, likely playing a role in the neurotoxic effect.

## 5. Conclusions

The phasor approach applied to confocal FLIM (FLIM-ϕ) provides an efficient and reproducible system for the measurement of membrane viscosity perturbation effects. Thanks to membrane-targeted probes, the implemented platform proved sensible to nanomolar concentrations of Aβ peptide.

On the one hand, the implications of these results extend to understanding the molecular mechanisms underlying membrane-associated toxicity under conditions mimicking Alzheimer’s disease, providing a potential target for therapeutic intervention. On the other hand, switching to an opposite perspective, we have also demonstrated that the system composed by a cell culture treated with a fluorescent probe and imaged by FLIM-ϕ can reveal the presence of Aβ in a biological fluid down to nanomolar concentrations. This feature perfectly matches the conventionally accepted definition of a “biosensor”, where the actual sensing element should be bio-derived [70]. As a proof of concept, these results suggest the potential for a non-invasive diagnostic tool where a properly calibrated sensing platform relying on cellular elements can detect the presence of Aβ (or other membrane order-affecting elements) in a biological sample. Indeed, the same approach could be extended to the detection of other molecular biomarkers able to affect the membrane order of cells, such as sphingolipids, cytokines, fatty acids, etc. The design of a biosensing platform for the early detection of AD could allow not only for timely diagnosis and intervention, potentially slowing down the disease progression and improving the patient’s quality of life, but also for evaluating potential drug candidates and understanding the disease pathways.

## Figures and Tables

**Table 1:** Average L_o_ fraction (±SD) for cells imaged by FLIM-ϕ under different treatment conditions.

	Control	Aβ-Olig 50 nM t = 1 h	Aβ-Olig 100 nM t = 0	Aβ-Olig 100 nM t = 1 h	Aβ-Fib 100 nM t = 0	Aβ-Fib 100 nM t = 1 h
**Average** **L_o_ fraction**	0.50 ± 0.05	0.54 ± 0.04	0.50 ± 0.02	0.59 ± 0.02	0.53 ± 0.03	0.62 ± 0.02

## Data Availability

The raw data supporting the conclusions of this article will be made available by the authors on request.

## Supplementary Materials

The following supporting information can be downloaded at: https://www.mdpi.com/article/10.3390/mi16020234/s1.
