# Supplementary material for: FLIM-Phasor Analysis (FLIM-ϕ) of Aβ-Induced Membrane Order Alterations: Towards a Cell-Based Biosensor for Early Alzheimer’s Disease Diagnosis
Source: Micromachines (Basel). 2025 Feb 19;16(2):234. doi: 10.3390/mi16020234 (PMC11857758; doi:10.3390/mi16020234)
Supplement: Supplementary file 1 [file micromachines-16-00234-s001.zip › micromachines-3443271-supplementary.pdf]

Supplementary materials

Article

FLIM-phasor analysis (FLIM-ϕ) of Aβ-induced membrane order alterations: towards a cell-based biosensor for early Alzheimer's disease diagnosis

Antonella Battisti [1,3*], Maria Grazia Ortore [2], Silvia Vilasi [3], Antonella Sgarbossa [1]

1 CNR – Nanoscience Institute, p.zza San Silvestro 12, I-56127 Pisa - Italy
2 Università Politecnica delle Marche, Dipartimento di Scienze della Vita e dell'Ambiente, via Brecce Bianche, I-60131 Ancona -Italy
3 CNR – Biophysics Institute, via Ugo La Malfa 153, I-90146 Palermo - Italy

* Correspondence: antonella.battisti@cnr.it

FLIM-ϕ principles outline

Fluorescence lifetime imaging (FLIM) can be coupled to the phasor analysis (FLIM-ϕ) to avoid issues related to a low number of collected photons per pixel and to fitting procedures. The transformation of fluorescence decays into phasors has been originally described by Jameson, Gratton *et al.*[1]. Acquisitions in the frequency domain ate typically performed with a continuous light source; however, pulsed excitation can be used as well by application of Fourier transforms exploiting the harmonic content of a repetitive pulsed source [2]. After the acquisition of a FLIM image with a TCSPC setup the $g_{i,j}(\omega)$ and $s_{i,j}(\omega)$ coordinates in the phasor plot corresponding to a fluorescence decay $I_{i,j}(t)$ collected from every pixel i,j can be obtained as in the following:

$$g_{i,j}(\omega) = \frac{\int_0^T I_{i,j}(t)\cos(\omega t)\,dt}{\int_0^T I_{i,j}(t)dt} \quad \text{(eq. S1)}$$

$$s_{i,j}(\omega) = \frac{\int_0^T I_{i,j}(t)\sin(\omega t)\,dt}{\int_0^T I_{i,j}(t)dt} \quad \text{(eq. S2)}$$

where ω, the laser repetition angular frequency (or the angular frequency of light modulation), is equal to $2\pi/T$, where T is the period of the laser pulses, and $I_{i,j}(t)$ is the intensity decay measured from time domain measurements.

Each pixel in the FLIM image becomes a single pixel in the phasor plot. Thanks to colored cursors, the SimFCS software (https://www.lfd.uci.edu/globals/) allows to select pixels in the phasor plot so

that the corresponding pixels in the FLIM image appear colored accordingly, composing a lifetime map. When the fluorescence decay corresponds to a double exponential due to the presence of two fluorescent species or of a single species with two well-distinguished photophysical states, the measured lifetimes are located along the segment between the phasors of the two main lifetime components and, since phasors follow the rules of vector algebra, it is possible to geometrically resolve the fractions of the two single components by the lever rule [3]. This keeps true even when the decays of the two single components are described by a multiexponential behaviour, because the Fourier transform is a linear operator. When two distinguishable optical states are present in a mixture, the mixture phasor coordinates can be written as:

$$g_{i,j}(\omega) = x_1 \cdot g_{i,j}(\omega)_1 + x_2 \cdot g_{i,j}(\omega)_2 \quad \text{(eq. S3)}$$

$$s_{i,j}(\omega) = x_1 \cdot s_{i,j}(\omega)_1 + x_2 \cdot s_{i,j}(\omega)_2 \quad \text{(eq. S4)}$$

where x_1 and x_2 are the molar fractions of states 1 and 2, respectively, *i.e.* the molar fraction-weighted sum of phasors (vectors) in the *g,s* plane. In this paper, the two components are represented by Ge1L fluorescence in a pure ordered and pure disordered phase [4], where it displays different lifetimes. The measured *g* and *s* values for Ge1L in pure L_o and L_d phases (taken as the coordinates of cursors encompassing the pixel clouds) are reported in Table S1 along with average lifetime values τ_{av}.

**Vesicle**	***g***	***s***	**τ_{av} (ns)**
L_o	0.33	0.46	5.03
L_d	0.64	0.46	2.69

Table S1: g, s and average lifetime (τ_{av}) values for the pure ordered (L_o) and disordered (L_d) phases.

Amyloid fibrils detection

The Aβ aggregation process was followed by light scattering measurements at 90° (λexc = λem = 405 nm). The 75 µM solution of Aβ1–40 in PBS was incubated at 45 °C under magnetic stirring in the thermostatic cell holder of a Fluoromax-4 spectrofluorometer (Horiba Jobin Yvon, Longjumeau, FR) to induce fibrillation, and light scattering was monitored for 6 hours. The first 3 hours of monitoring, revealing the gradual increase in aggregates size, are reported in fig. S1. After 3 hours, the curve reaches a plateau and no further increase occurs.

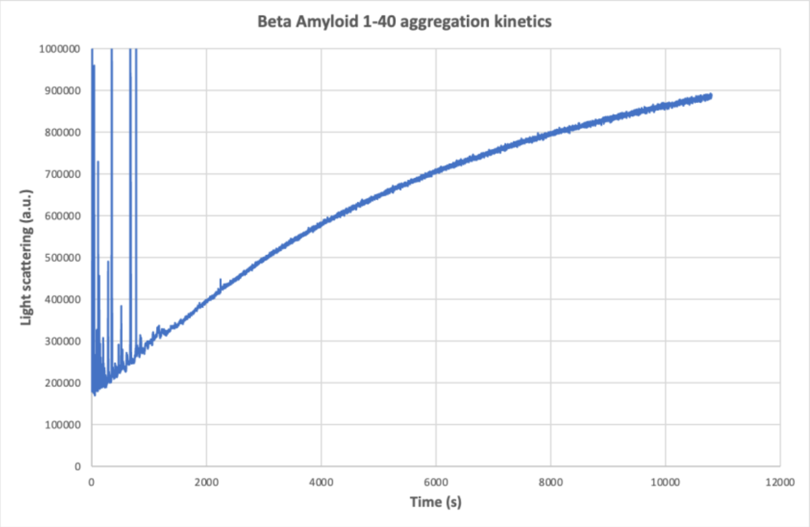

Figure S1: Aggregation kinetics of Aβ$_{1\text{-}40}$ followed by light scattering at 405 nm.

The thioflavin T (ThT) test was performed on amyloid samples at the beginning and end of the aggregation kinetics to verify the formation of fibrils [5]. Briefly, 5 μM ThT was added to 75 μM Aβ$_{1\text{–}40}$ in 0.1 M PBS (pH 7.4) as prepared (t=0) or after 6 h of incubation at 45 °C (t=6 h) and the resulting fluorescence was measured in the fluorometer, with λ_{exc} = 442 nm and a detection range between 450 and 700 nm. The appearance of a fluorescent peak at 490 nm reveals the binding between ThT and amyloid fibrils (fig. S2).

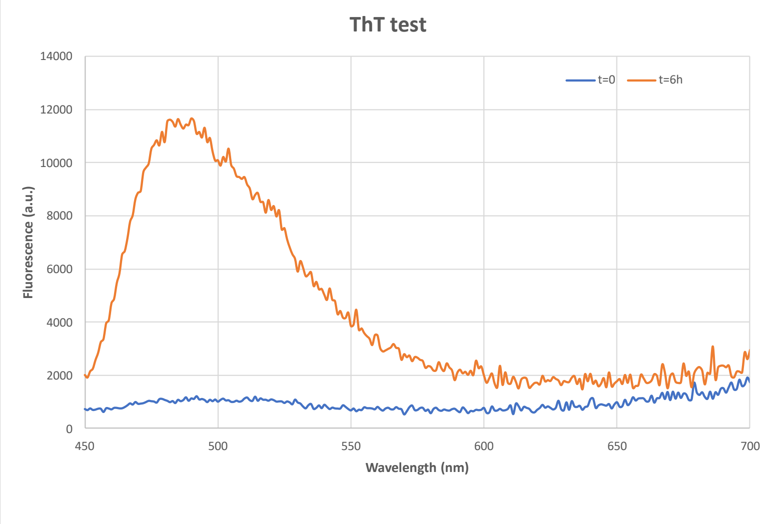

Figure S2: ThT fluorescence in Aβ solutions at t=0 (blue) and t=6 h (red).

Additionally, the ThT-stained aggregates were imaged using a confocal fluorescence microscope (Leica TCS SP5, Leica Microsystems AG, Wetzlar, Germany). Surrounded by a uniform background fluorescence due to smaller aggregates, large fibril bundles were identified as well as micrometric fibrillar structures (fig. S3).

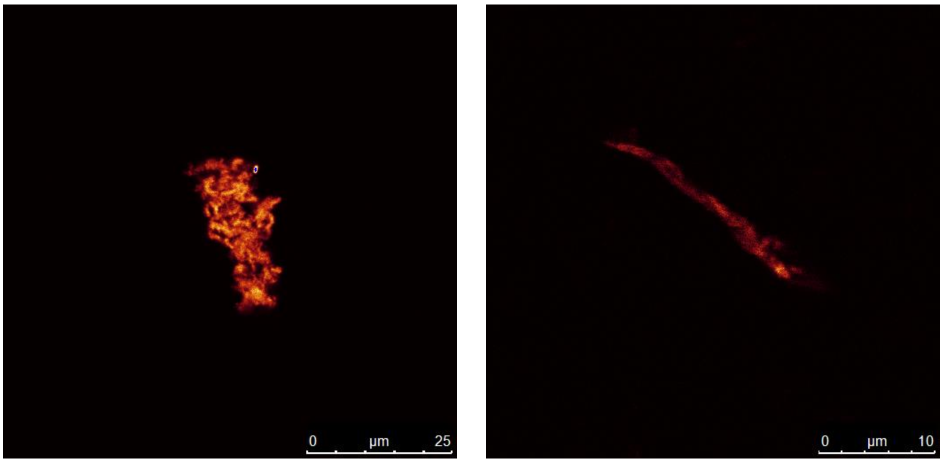

Figure S3: Confocal imaging of micrometric fibrillar bundles (left) and of a single micrometric fibril (right).

References

1. Jameson, D.M.; Gratton, E.; Hall, R.D. The Measurement and Analysis of Heterogeneous Emissions by Multifrequency Phase and Modulation Fluorometry. *Appl Spectrosc Rev* **1984**, *20*, 55–106, doi:10.1080/05704928408081716.
2. Malacrida, L.; Ranjit, S.; Jameson, D.M.; Gratton, E. The Phasor Plot: A Universal Circle to Advance Fluorescence Lifetime Analysis and Interpretation. *Annu Rev Biophys* **2021**, *50*, 575–593, doi:10.1146/ANNUREV-BIOPHYS-062920-063631/CITE/REFWORKS.
3. Digman, M.A.; Caiolfa, V.R.; Zamai, M.; Gratton, E. The Phasor Approach to Fluorescence Lifetime Imaging Analysis. *Biophys J* **2008**, *94*, L14, doi:10.1529/BIOPHYSJ.107.120154.
4. Abbandonato, G.; Storti, B.; Tonazzini, I.; Stöckl, M.; Subramaniam, V.; Montis, C.; Nifosì, R.; Cecchini, M.; Signore, G.; Bizzarri, R. Lipid-Conjugated Rigidochromic Probe Discloses Membrane Alteration in Model Cells of Krabbe Disease. *Biophys J* **2019**, *116*, 477–486, doi:10.1016/J.BPJ.2018.11.3141.
5. Xue, C.; Lin, T.Y.; Chang, D.; Guo, Z. Thioflavin T as an Amyloid Dye: Fibril Quantification, Optimal Concentration and Effect on Aggregation. *R Soc Open Sci* **2017**, *4*, 160696, doi:10.1098/RSOS.160696.
